# Experimental and numerical study of the mixed lubrication under the action of magnetic ionic liquid additives

**DOI:** 10.1038/s41598-024-55607-3

**Published:** 2024-03-07

**Authors:** Ze Liu, Zhijun Yan, Shibo Wu, Haocheng Sun, Shengwei Zhang

**Affiliations:** Marine Engineering College, Dalian Maritime University, Dalian, 116026 Liaoning China

**Keywords:** [bmim][FeCl_4_], Boundary film strength model, Mixed lubrication, Fluid dynamics, Mechanical engineering

## Abstract

In this paper, the tribological characteristics of an oil-soluble magnetic fluid additive under mixed lubrication are studied by experiments and numerical simulation. [bmim][FeCl_4_] is dissolved in CF10W-40 lubricating oil as a magnetic liquid additive, and its friction coefficient is tested by a point contact friction tester at different temperatures, rotational speeds and magnetic field intensities. The transition condition of lubrication state is obtained through analyzing the Stribeck curves based on the experiments, and the strength model of boundary film is established accordingly. A mixed lubrication model is established by substituting the boundary film strength model and the surface roughness model into the hydrodynamic lubrication model based on Reynolds equation. The results show that the magnetic solution as an additive can obviously reduce friction and wear, and the effect is more obvious under the condition of magnetic field. The boundary film strength model can accurately reflect the transition characteristics of lubrication state in the presence of boundary film, and the mixed lubrication model based on boundary film strength model can more precisely reflect the tribological characteristics of friction pairs, so this study provides a new theoretical method for the related research on the influence of boundary film on lubrication characteristics.

## Introduction

With the continuous progress of industrial technology, the performance requirements for lubricants are becoming more and more stringent. Tribology pairs should not only have high strength bearing capacity, good anti-friction and anti-wear resistance effects but also show self-repair ability and environmental adaptability. In this regard, magnetic liquid as a new type of lubricant has good application significance. Under the action of the external magnetic field, the magnetic liquid lubricant can repair the scratches on the friction surface, and can also offset the gravity and centripetal force in the lubrication stage to prevent leakage and environmental pollution^1^. Therefore, magnetic liquids are essential materials for solving lubrication and sealing problems of moving parts of mechanical equipment under specific working conditions. Magnetic liquids are usually composed of nanoparticles dispersed in the lubricating base fluid. However, the application conditions and service life of such magnetic lubricants are limited due to the agglomeration of nanoparticles and poor suspension dispersion. Therefore, there is a need to explore new magnetic lubricant materials^2^.

Magnetic ionic liquids are thermodynamically stable homogeneous systems. Unlike conventional magnetic liquids, magnetic ionic liquids do not suffer from problems such as nanoparticle agglomeration and sedimentation, so they can accurately fill the contact surfaces for continuous lubrication. Magnetic ionic liquids can also be magnetized by the external magnetic field to produce a certain response due to the presence of an anion in the magnetic center of the magnetic ionic liquid, which is formed by a single-electron organic structure or a complex structure containing metal ions^3^. Therefore, magnetic ionic liquids have special significance in tribology^4–6^. Driven by these ideas, some researchers have been studying the synthesis of new MILs and studying their physical and chemical properties^7,8^. Jia et al.^2^ prepared [C6mim]5[Dy(SCN)8] as a lubricant for steel-steel friction pairs, and proved by experiments that the ionic liquid produced a boundary film containing iron elements on the surface of the friction pair in the experiment, which was better than the nano-magnetic fluid anti-friction and anti-wear effect.

In order to better understand the contact mechanism under various working conditions, predict the lubrication state, and improve the reliability of the part design, it is crucial to establish theoretical studies and mathematical models of mixed lubrication. As early as 1886, Reynolds^9^ proposed the Reynolds equation, which provided the basis for fluid lubrication and is the core equation for dealing with lubrication problems. In 1972, Johnson and Greenwood^10^, in their mathematical derivation of the mixed lubrication model, first proposed that the normal external load be shared by the asperity and the lubricant film. In 2000, Hu and Zhu^11^ proposed to solve the fluid and contact zone pressures simultaneously by the unified Reynolds equation, which is currently the most used model. In 2015, Masjedi and Khonsari^12^ used statistical methods to simulate asperity contact and proposed a more accurate mixed lubrication model.

Due to the calculation formulas of oil film thickness and roughness load in numerical calculation are related to surface roughness, the effect of surface roughness on friction and wear is complex. Researchers investigated the effect of the surface roughness on friction in different lubrication states. Wang et al. revealed that surface roughness has a greater influence on friction coefficient in mixed lubrication than in fluid lubrication and boundary lubrication. Dong et al.^13^ found that the rougher the friction surfaces are, the easier it is to enter boundary lubrication. Moreover, there is an optimal surface roughness to achieve the minimal friction coefficient for each lubrication state. Jeng et al.^14^ pointed out that the surface roughness significantly influences the friction force in thin film lubrication. With the lubricant film thickness decreasing, the influence of the surface roughness becomes increasingly significant. Liu et al.^15^ concluded that the real-time wear rate is positively correlated with the real-time surface roughness. Pan et al.^16^ conclude when the surface roughness decreases further and passes the critical point, the asperities cannot fully store the wear debris, resulting in severe abrasive wear and an increase in the plowing force. Consequently, friction failure occurs, increasing the friction coefficient and the wear rate.

In the Reynolds equation, the major input parameters for the lubricant are viscosity and density. However, when additives are present or other conditions are introduced, the additives affect the oil film properties at constant viscosity, and lubricant film failure does not necessarily occur even if the oil film becomes very thin. Boundary film strength model can be used to determine whether the lubricant film is failure or not. Therefore, to more accurately assess the lubrication condition as well as the coefficient of friction, the effect of boundary film properties needs to be considered in mixed lubrication. In 1939, Blok^17^ proposed that when the contact flash temperature reached a critical value, the surface would be glued to cause the boundary film to fail. In 2012, Yan and Yang^18^ regarded temperature as the criterion for the failure of boundary lubrication film, and established the failure model of boundary film. In 2016, Zhang and Spikes^19^ confirmed that the shear force has a serious effect on the failure of the boundary film. Additionally, in 2018, Savolainen and Lehtovaara^20^ showed that the thermal effect played a key role in the failure of the surface boundary film, and there is a critical temperature that caused the failure of the boundary film. In 2019, Lee et al.^21^ derived a boundary film failure model based on the principle of additive mass conservation and considered that the failure of boundary film was caused by the depletion of additives during sliding. In 2021, according to the thermal fluid film thinning effect, Lyu et al.^22^ suggested that the higher shear energy of fluid would lead to the failure of boundary film. In 2023, Zhang^23^ proposed a boundary film failure model that comprehensively reflects the influence of shear force and temperature rise. The model can be used to determine whether the boundary film fails under certain conditions.

Under the action of magnetic field, the magnetic fluid can be adsorbed on the surface of the friction pair to form a tight surface film and improve the strength of the boundary film. However, the single hydrodynamic fluid lubrication and the asperity contact model cannot accurately reflect the improvement effect of the boundary film lifting on the lubrication state. The boundary film strength model can accurately reflect the characteristics of the boundary film in mixed lubrication. Therefore, a boundary film strength model considering magnetic field conditions is proposed in this paper, and the model is coupled with the hydrodynamic fluid lubrication model and the asperity contact model to obtain a mixed lubrication model that can accurately reflect the mixed lubrication characteristics under the action of magnetic fluid. In this paper, the boundary film strength model under the action of magnetic ion solution additive is fitted by the results of the experimental test. The model can be used to evaluate the influence of line contact friction pairs on lubrication performance under different working conditions, such as speed, viscosity, and surface roughness under the action of magnetic ion liquid, and provide an excellent theoretical basis for the application of magnetic ion liquid additives to practical mechanical friction pairs.

## Magnetic ionic liquid additive

### Introduction of magnetic ionic liquid additive ([bmim][FeCl_4_])

**Figure 1:**
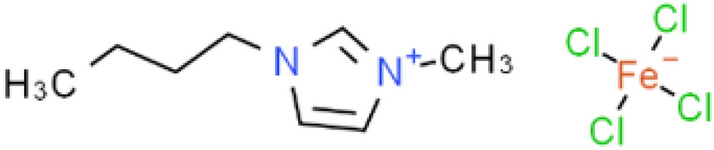
The structure of [bmim][FeCl_4_].

Figure 1 illustrates the structure of [bmim][FeCl_4_]. The magnetic property of the ion liquid is due to the presence of magnetic centers in the tetrachloroferrate anion FeCl_4_^−^, which can respond to the magnetization of the external magnetic field. In a previous paper, we reported that 1-butyl-3-methylimidazolium tetrachloroferrate ([bmim][FeCl_4_]), exhibited an unexpectedly strong response to a magnet^8^. Table 1 shows the main characteristic parameters of [bmim][FeCl_4_]. The density of [bmim][FeCl_4_] is obtained by calculating the ratio of the mass (measured by electronic balance) to volume (measured by graduated cylinder).The magnetic susceptibility of [bmim][FeCl_4_] is measured using an MPMS SQUID measuring system.

**Table 1:** Parameters of [bmim][FeCl_4_].

Parameter	Value
Temperature	26.85 °C
Density	1.31 g/mL
Molar susceptibility	0.0137 emu/mol

**Figure 2:**
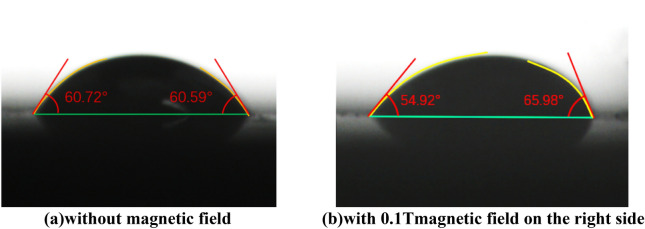
The results of contact angle measurement.

In Fig. 2, the contact angle of the ionic liquid is measured by JC2000D contact angle measuring instrument both with and without a magnetic field. When using the contact angle measuring instrument, the drop of each drop is set to 5 μL. The magnetic field condition is realized by adding a permanent magnet, and the magnetic field intensity of the droplet is measured by the TD8650 digital Tesla magnetic tester. Figure 2a shows the contact angle of [bmim][FeCl_4_] without the magnetic field, while Fig. 2b shows the contact angle of [bmim][FeCl_4_] with a horizontal magnetic field of 0.1 T applied on the right side. The figures suggest that the presence of a magnetic field affects the contact angle. Specifically, the contact angle of the droplet in the right figure (b) is larger than that in the left figure (a), and this change in contact angle indicates that the ionic liquid can respond to the magnetic field. This is due to the response of FeCl_4_^−^ to the magnetic field force after being magnetized by the magnetic field. Under the action of the magnetic field, the magnetic field force along the direction of the magnetic field (horizontal to the right) makes the ionic liquid move to the right, so that more magnetic ions are accumulated on the right side of the droplet, resulting in a larger contact angle on the right side.

### Preparation of magnetic fluid

**Figure 3:**
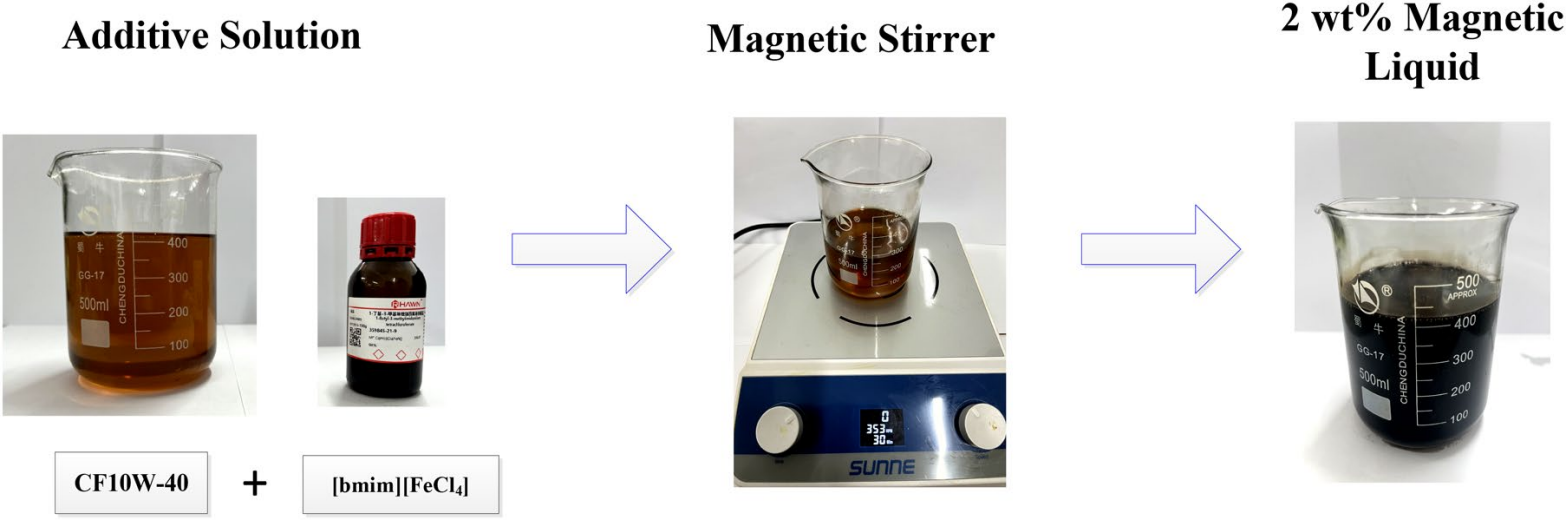
Configuration flow chart.

After multiple sets of tests, it is found that the mass fraction of the configured magnetic fluid is too low, the response to the magnetic field is not obvious, and the anti-friction and anti-wear effect is not good. When the mass fraction of the configured magnetic fluid is too high, the cost is high and the further improvement of anti-friction and anti-wear effects are not significant. Therefore, the magnetic fluid with a mass fraction of 2% is finally selected. The 8 g [bmim][FeCl_4_] ionic liquid is added to a beaker containing 392 g CF10W-40 lubricating oil. To make the [bmim][FeCl_4_] ionic liquid better dissolve in the lubricating oil, the mixed reagent is placed in a magnetic stirrer and stirred at a speed of 360 rpm/min for 30 min to obtain a magnetic liquid with a mass fraction of 2%. The operation process is shown in Fig. 3.

## Friction and wear tests

### Test condition

**Figure 4:**
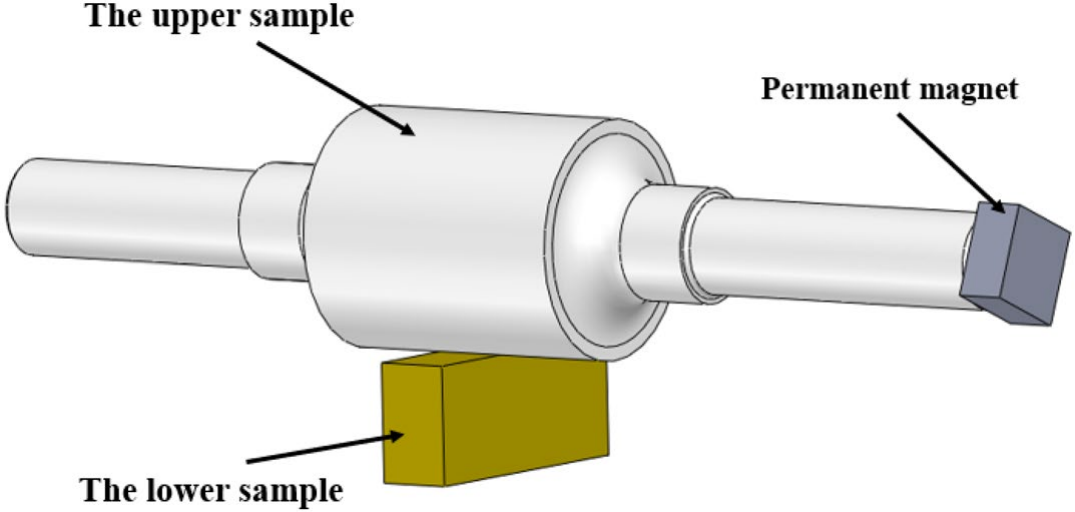
Schematic diagram of tests.

The test selected a 45-steel stepped shaft as the upper specimen, and the machining accuracy is IT5. The cylindrical surface of the middle part of the stepped shaft is the test working face, with a diameter of 70 mm. The lower specimen is made of an H59 brass metal block, the working surface size is 10 mm × 60 mm, and the comprehensive surface roughness Ra of two surfaces is 0.11 μm. The magnetic field condition of the test is provided by a 46 mm × 46 mm × 21 mm N38 square magnet, as shown in Fig. 4. The test temperature and lubricant viscosities are shown in Table 2.

**Table 2:** Parameters of test.

Parameter	Value
Ambient temperature	18 °C
Lubricant viscosity of CF10W-40	0.16 Pa·s
Lubricant viscosity of magnetic liquid	0.197 Pa·s
Lubricant viscosity of magnetic liquid in 0.1T magnetic field	0.198 Pa·s

As an imidazole ion solution, [bmim][FeCl_4_] is composed of positively charged imidazole ions and negatively charged ferric chloride ions. When the imidazole ion solution is mixed with the lubricating oil, the positive charge of the imidazole ion will attract the negative charge region in the lubricating oil, and the negative charge of the anion will attract the positive charge region in the lubricating oil. This attraction will aggregate the lubricating oil molecules, increasing the viscosity of lubricating oil.

### Test results

**Figure 5:**
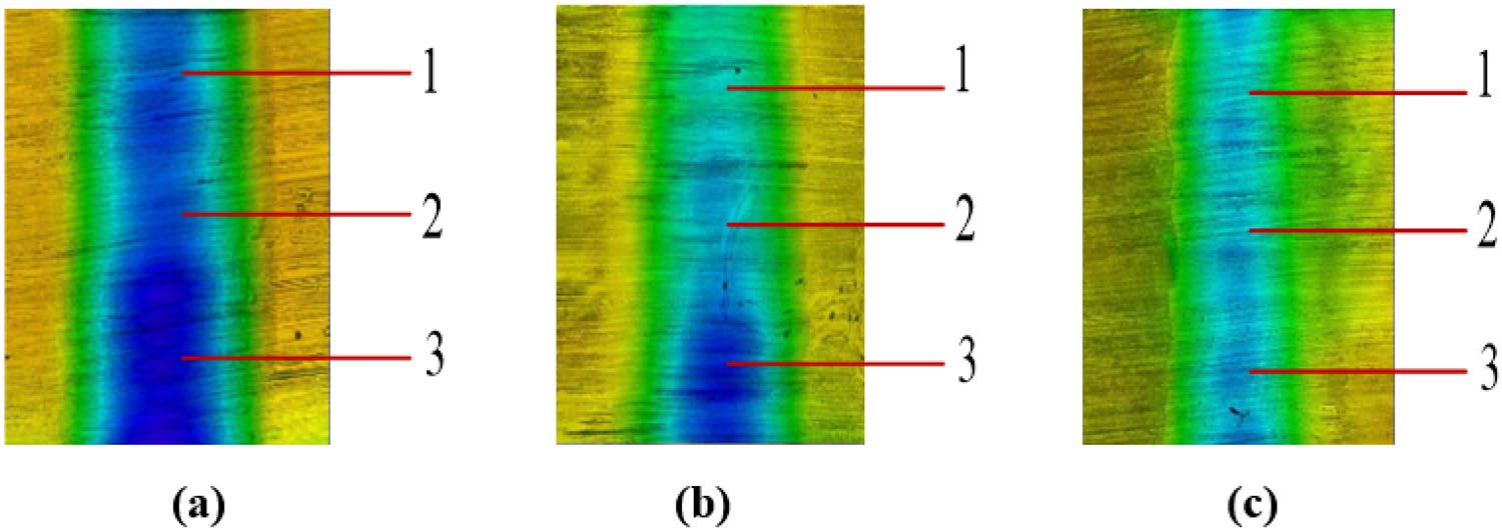
White light interference test diagram.

**Figure 6:**
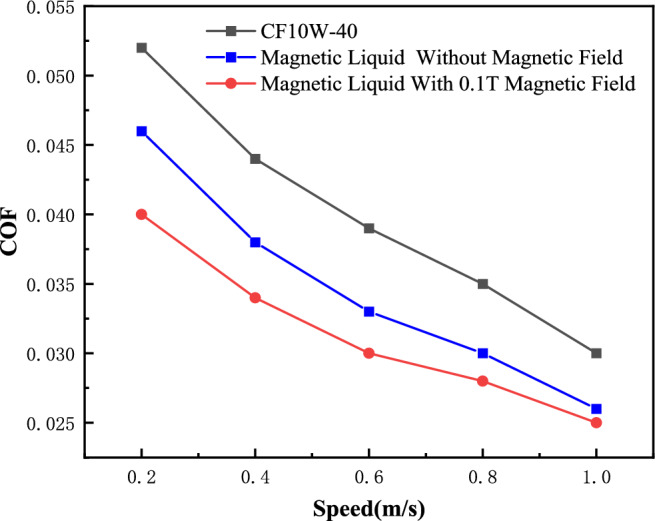
The friction coefficient comparison diagram of different conditions.

The wear test is performed using a line contact wear test machine under three different conditions: CF10W-40 lubricating oil, magnetic fluid lubricating oil, and magnetic fluid lubricating oil with a 0.1 T magnetic field. The test is conducted with a loading force of 1800 N, speed of 0.4 m/s, and duration of 150 min. Afterward, the specimens are observed under a white light interferometer to examine their wear marks. To ensure accuracy, three points (1, 2, and 3) are taken from top to bottom during the inspection. The results are shown in Table 3 and Fig. 5. Based on the test results, the wear depth is found to be the highest when using CF40 lubricating oil. However, when using magnetic fluid lubricating oil, the wear depth decreases by approximately 45%, indicating a noticeable anti-wear effect. The addition of magnetic field conditions further reduces the anti-wear impact under magnetic fluid conditions. Additionally, the friction coefficient is tested under varying speed conditions with and without a magnetic field. The results are shown in Fig. 6. When subjected to an external magnetic field, magnetic fluid exhibits. About 10% friction reduction effect and about 20% wear reduction effect.

**Table 3:** Wear depth (μm).

Test condition	1	2	3
CF10W-40	11.584	12.285	13.023
Magnetic liquid	5.385	6.801	7.362
Magnetic liquid + 0.1 T magnetic field	4.279	4.434	4.701

**Figure 7:**
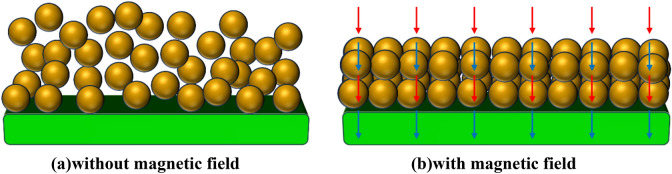
The schematic diagram of magnetic ion movement under magnetic field conditions.

The magnetic ion liquid has high polarity, which makes it easy to adhere to metal surfaces and form a boundary film. It is also paramagnetic, which means it can respond to magnetic fields. When an external magnetic field is applied, the spin direction of magnetic ions becomes aligned with the direction of the magnetic field, which makes the arrangement of the ions more stable and orderly. Therefore, the magnetic ion solution can produce a thick boundary film on the surface of the friction pair, and the strength of the boundary film is also enhanced. Under the condition of no external magnetic field, ionic liquid can also improve the stability of oil film. On the one hand, the particles are free to move randomly due to thermal motion^24^, as shown in Fig. 7. On the other hand, the cations and anions in the ionic solution attract each other to make them close together. But at a certain distance, these species go far from each other due to the direct repulsive force between the nucleus and the nucleus. Scholars such as Azadeh Daneshvar, Majid Moosavi and Hassan Sabzyan have explained this phenomenon^25^. They called this phenomenon breathing mode of the box and makes wavelength in the number density plots. The breathing mode of the box results in the ion solution having a specific wavelength in terms of its number density. The presence of the magnetic field further stabilizes the number density wavelength of the ion solution, making the anions and cations reduce fluctuations in the breathing mode. The above actions enhance the stability of ionic solution and help the oil film bear stronger shear stress.

As shown in Table 2, the viscosity of magnetic fluid lubricating oil is increased by about 0.5% under the magnetic field condition (as shown in Table 2). At present, the contribution of the increase of lubricating oil viscosity and boundary film strength to the improvement of friction reduction and wear resistance under magnetic field conditions is still unclear. Therefore, it is necessary to study the boundary film strength of magnetic liquid under magnetic field condition.

## Boundary film strength model

### Test equipment and test preparation

**Figure 8:**
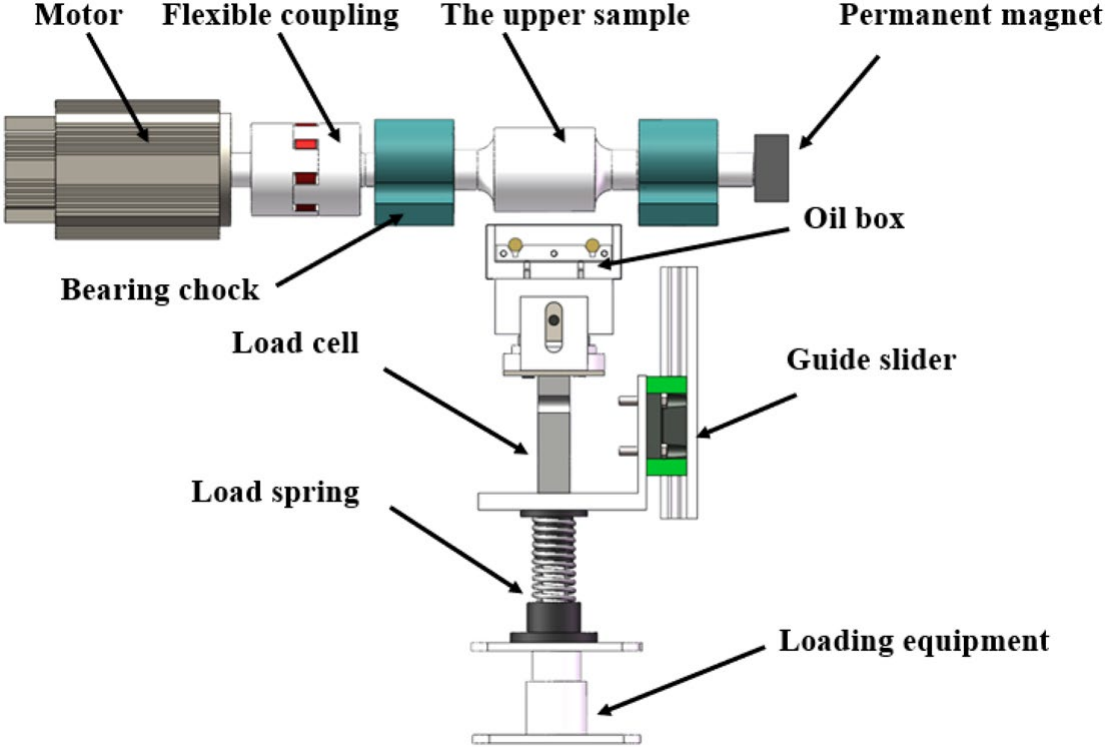
Schematic diagram of double-point contact friction test machine.

**Figure 9:**
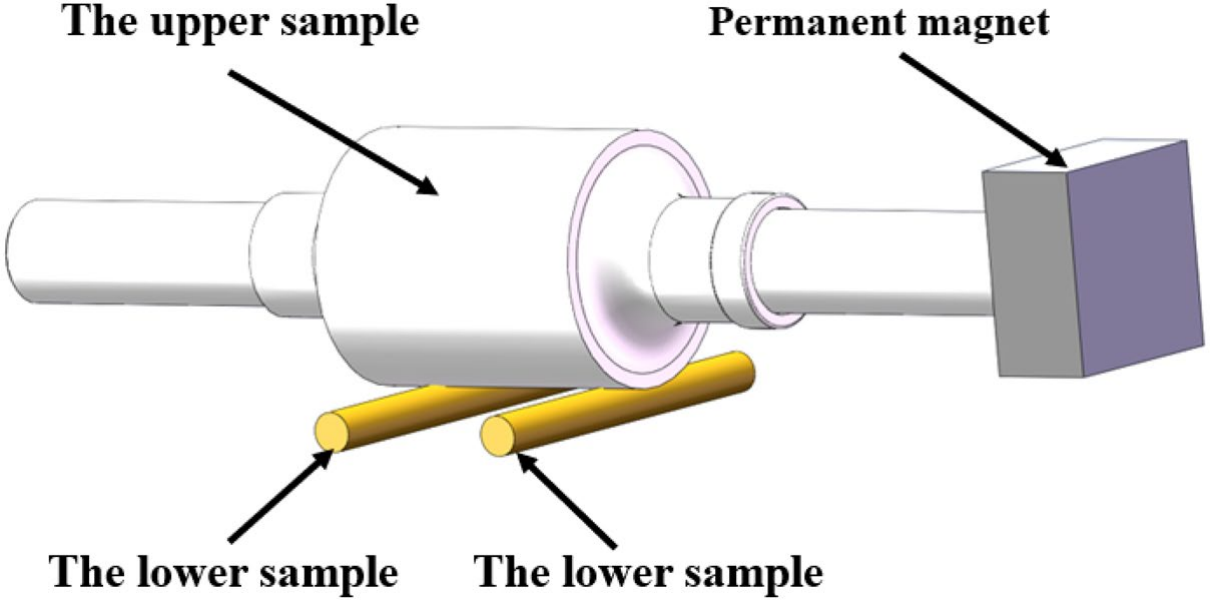
Schematic diagram of friction pair.

In this study, a self-made double-point contact friction test machine has been designed to test the boundary film strength. Figures 8 and 9 show the structure of the test machine and the sample installation.

The material of the upper sample in the double-point contact friction test machine is a stepped shaft of 45 steel (ISO standard steel number C45E4). The work face is a cylindrical surface with a diameter of 50 mm, and the machining accuracy is IT5. The lower sample is a cylindrical copper rod made of H59, and the test face is a cylindrical surface with a diameter of 8 mm. The comprehensive surface roughness is 0.59 μm. Table 4 shows the characteristic parameters of the samples.

**Table 4:** Characteristic parameters of the samples.

Parameter	Value
Radius of the upper sample,* R*_*x*_	0.025 m
Radius of the lower sample,* R*_*y*_	0.004 m
Equivalent elastic modulus, *E*	148 GPa
Surface roughness,* σ*	0.59 μm

**Figure 10:**
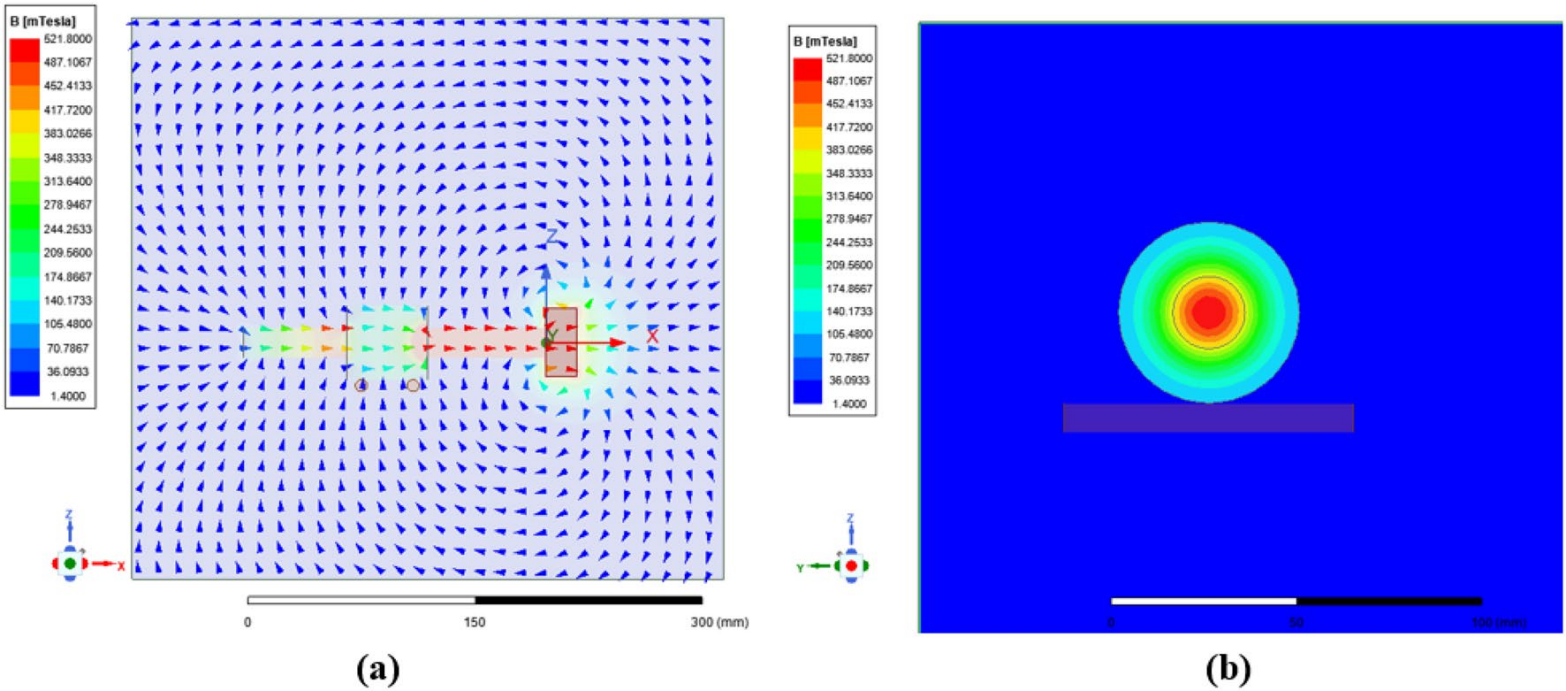
Simulation results of magnetic induction line direction.

The magnetic field condition is realized by adding N38 permanent magnet. In order to clarify the strength and direction of the magnetic field provided by the 50 × 50 × 30 mm size N38 permanent magnet for the testing machine, Maxwell software is used for simulation. In the simulation process, the same material and size as the upper and lower specimens in the test process are selected. The distribution of magnetic field intensity and magnetic induction line is simulated by Maxwell, as shown in Fig. 10. The axially distributed magnetic field can be obtained, that is, the horizontal magnetic field distributed from left to right on the working surface of the upper sample. Due to the difference in the size of the upper sample in the boundary film strength tests and the friction and wear tests in 2.3, it is necessary to control the magnetic field on the contact surface by controlling the size and quantity of the permanent magnet. The size of the magnetic field strength is verified by using Maxwell to provide a magnetic field of 0.1 T intensity.

### Test procedure

Before the test, the lower sample is placed in anhydrous ethanol for ultrasonic cleaning, and then the lower sample is fixed in the oil box, and the lubricating oil is poured into the oil box to submerge the lower sample completely. The permanent magnets is fixed on the upper specimen to provide axial magnetic field strength; the lubricating oil temperature is realized by the heating resistance in the oil box, and the temperature is controlled at the temperature required for the test. The test machine is first adjusted to the required speed and a lower loading force (20 N) and run continuously for 20 min to facilitate the formation of the boundary film. Then, the test machine is adjusted to the required speed and load to start the test. After stabilization, the average loading force and friction coefficient within 30 s under the modified working condition are measured. After each test, the position of the contact surface of the upper and lower sample is adjusted before the next test. Each working condition needs to be repeated three times to reduce the randomness. After each test, both the test sample and lubricating oil are replaced, and the oil box is cleaned.

### The failure features of boundary lubrication

Using the aforementioned testing machine, the changes of friction coefficient with load at five temperatures (35, 55, 80, 100 and 120 °C), different speeds (0.1, 0.3, 0.5, 0.75 and 1.0 m/s) and different magnetic field intensities (0, 0.05 and 0.1 T) are measured.

**Figure 11:**
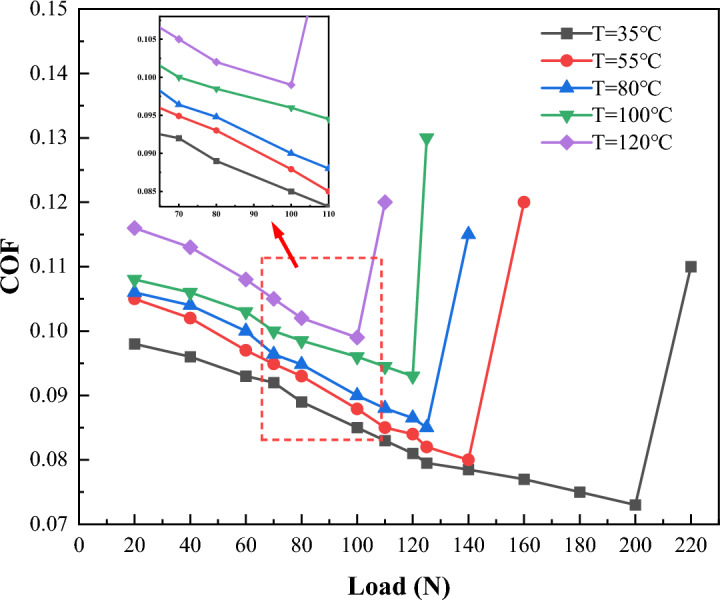
Variations of friction coefficient with temperature.

Figure 11 shows the variation of friction coefficient with load at different temperatures at 0.3 m/s speed under 0.1 T magnetic field strength. As the load continues to increase, there is a sudden increase in the friction coefficient when a certain load is reached. This sudden change indicates the change in the lubrication state. When the load increases the value corresponding to this point, the boundary film breaks, resulting in a sudden increase in the friction coefficient. With the increase in temperature, the boundary film fails under a lower load. When the temperature is 35 °C, the critical load of oil film failure is 200 N, and when the temperature is 120 °C, the critical load of oil film failure is 100 N. It can be seen that temperature has a significant effect on the failure of boundary film strength.

**Figure 12:**
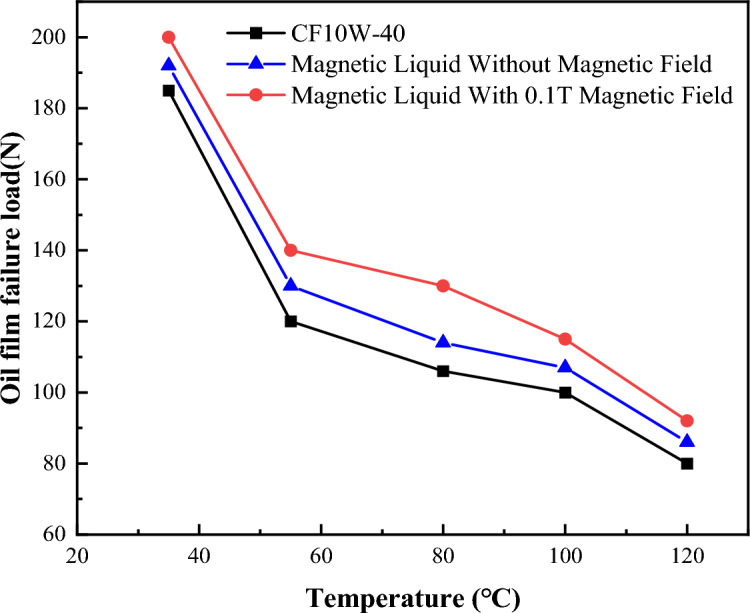
Schematic diagram of oil film failure load changing with temperature.

Figure 12 shows the critical loads of boundary film failure of CF10W-40 lubricating oil, magnetic liquid without a magnetic field and magnetic liquid with a magnetic field at different temperatures. It can be seen from the figure that with the increase of temperature, the critical oil film bearing capacities under the above-mentioned three conditions decrease nonlinearly. Below 60 °C, with the increase of temperature, the critical oil film bearing capacities decrease rapidly; Above 60 °C, with the increase of temperature, the downward trend of critical oil film bearing capacities slow down. The failure critical load of the boundary film of the magnetic liquid (dissolve [bmim][FeCl_4_] in CF10W-40 lubricating oil) is larger than that of the CF10W-40 lubricating oil at the same temperature and speed, it shows that magnetic liquid can withstand greater ultimate shear stress. Compared with the condition without magnetic field, the failure critical loads of the magnetic fluid lubricating oil under the magnetic field condition are larger, showing that the magnetic field can further improve the effect of its shear resistance.

**Figure 13:**
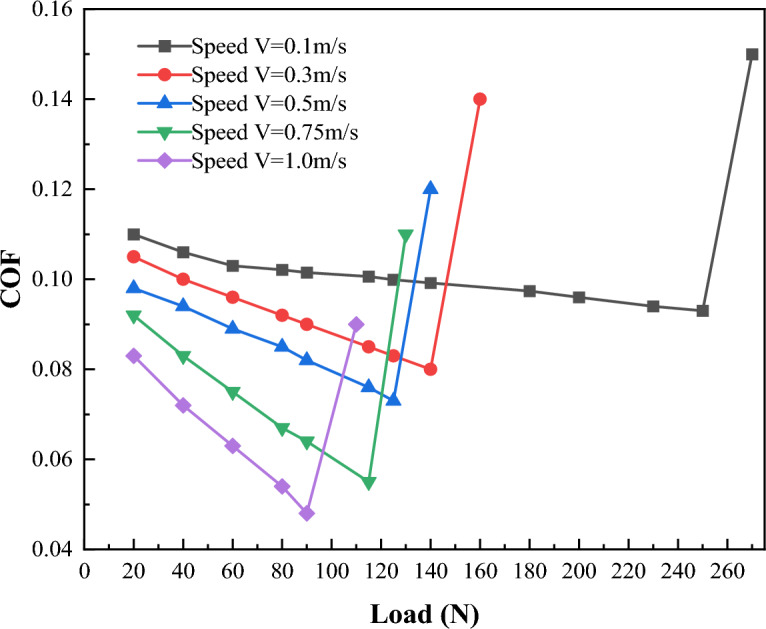
Variation of friction coefficient with sliding speed.

Figure 13 shows the variation of friction coefficient with load at different sliding speeds at 55 °C under 0.1 T magnetic field strength. As can be seen from the figure, corresponding to a specific sliding speed, with the increase of load, the friction coefficient will experience a sudden increase, which reflects the change of lubrication state from boundary lubrication state to dry friction state. The figure also reveals that with the increase of sliding speed, the critical load values corresponding the turning points decreases. For example, when the speed is 0.1 m/s, the inflection point of friction coefficient is 250 N, indicating that the critical load is 250 N; When the speed is 1 m/s, the critical load is 90 N. The reason for this phenomenon is that under the condition of boundary lubrication, with the increase of speed, the boundary film is more likely to rupture due to the greater shear force.

**Figure 14:**
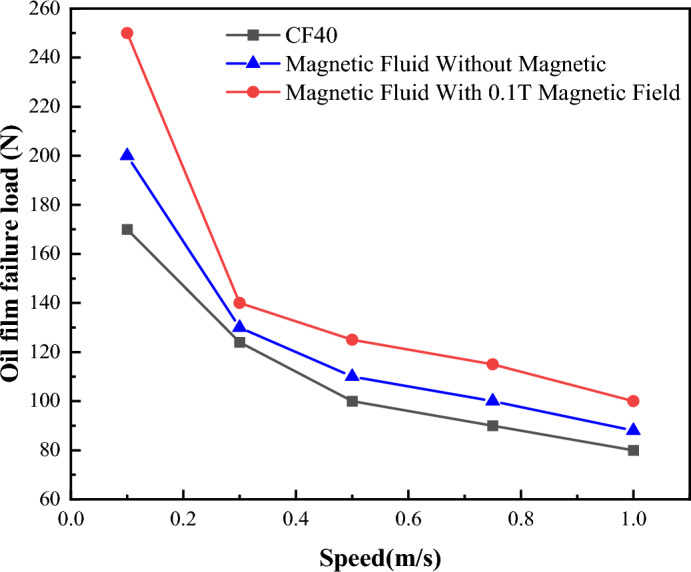
Schematic diagram of oil film failure load changing with sliding speed.

Figure 14 shows the critical load of boundary film failure of CF10W-40 lubricating oil, magnetic liquid without a magnetic field and magnetic liquid with a magnetic field under different speeds at 55 °C. It can be found that the lubricating oil with the addition of magnetic ion solution as an additive has a larger oil film failure load and can withstand greater shear stress than the non-additive lubricating oil. This is especially obvious at lower speeds. For example, compared with the condition without magnetic field, when the rotating speed is 0.1 m/s, the critical load value of boundary film under the condition of applying magnetic field increases by about 40%.

### The establishment of boundary film strength model

**Figure 15:**
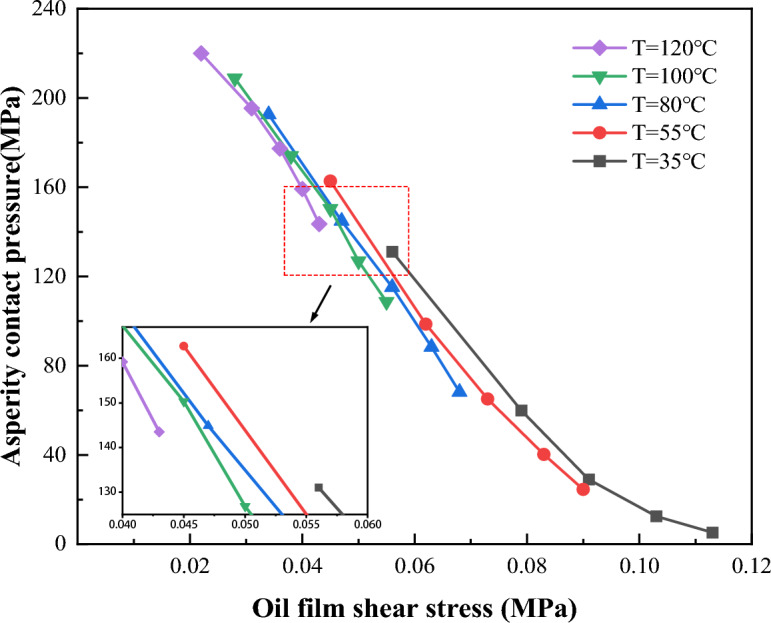
Relationship between asperity contact pressure and shear stress at different temperatures.

Corresponding to the working conditions corresponding to the boundary film failure in section “Test condition”, the oil film thickness h under each working condition is obtained according to Hamrock–Dowson point contact oil film thickness formula^26^, and the obtained oil film thickness is substituted into Ree-Eyring constitutive equation^27^ to calculate the oil film shear stress τ under each working condition. Then, according to G–T asperity contact formula^28^, the critical asperity contact pressure P_a_ when the boundary film fails is obtained. According to the calculation results, the relationship between the critical asperity contact pressure and the oil film shear stress of the boundary film failure at different temperatures under 0.1 T magnetic field strength is drawn as Fig. 15. It can be seen from the Fig. 15 that the contact pressure under the boundary film failure is 165 N when the shear stress is 0.045 MPa at 55 °C, and the contact pressure of the boundary film failure is 130 N when the shear stress is 0.09 N. At the same temperature, the greater the shear stress on the oil film, the smaller the oil film failure load is. When the temperature is 55 °C and the shear stress is 0.05 MPa, the oil film failure load is 150 N; when the temperature is 100 °C, the oil film failure load is 122 N. It can be seen that under the same shear stress conditions, the higher the temperature, the smaller the oil film failure load is.

**Figure 16:**
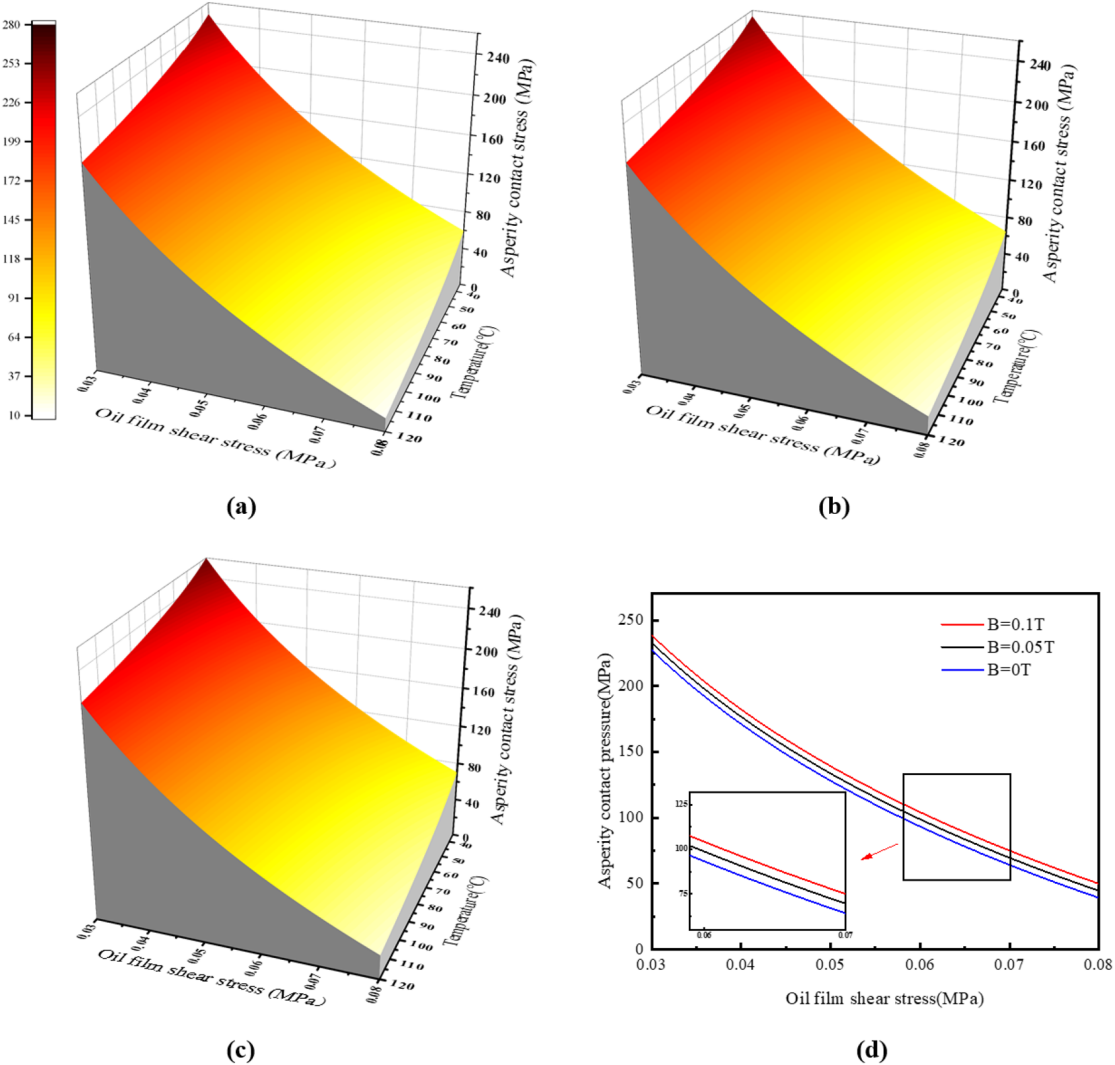
Model curved surface of boundary film strength.

From the above analysis, it can be seen that the boundary film failure under different magnetic field strengths *B*(T) is mainly related to the contact pressure *Pa* (MPa), temperature* T* (°C), and shear stress *τ* (MPa). Therefore, the boundary film strength model under magnetic field can be expressed as a three-dimensional curved surface as shown in Fig. 16, which can be used to judge whether the boundary film is invalid. In the picture (a), (b) and (c) depict three-dimensional images under 0 T, 0.05 T and 0.1 T magnetic field conditions, respectively, and Fig. 16d shows the parabolic comparison of the boundary film strength images under three different magnetic field strengths at 60 °C. When the working condition corresponds to the position below the curved surface, the boundary film will not fail; However, when the working condition corresponds to the position above the curved surface, it indicates that the boundary film is broken. By fitting the experimental data with the calculated results, the boundary film strength model considering the magnetic field strength is obtained as follows.

1 $$ Pa = S({\text{B}},{\text{T}},\tau ) = 3280 \times {\text{e}}^{0.031 \times B} \times {\text{T}}^{ - 0.01045} \times \tau^{ - 0.05216} - 3546 $$

## Establishment of mixed lubrication model

In order to study the influence of magnetic ionic solution as an additive on the anti-friction and anti-wear performance of practical friction pairs, a mixed lubrication model of magnetic ion solution as an additive lubricating oil under magnetic field conditions is established. The accurate mixed lubrication model is inseparable from the application of the boundary film failure model. Therefore, the boundary film strength model obtained in section “Boundary film strength model” is coupled with the asperity contact model and the hydrodynamic lubrication model to establish a mixed lubrication model considering the boundary film strength. Under the condition of mixed lubrication, the load is shared by the lubricant film and the asperity, and the contact pressure *P* in the contact area is the sum of the oil film pressure* P*_*h*_ and the asperity contact pressure *P*_*a*_:

2 $$ P = P_{h} + P_{a} $$

### Lubrication model

For the cylindrical surface and plane linear contact friction pair shown in Fig. 4, the following one-dimensional linear contact Reynolds equation is adopted.

3 $$ \frac{{{\text{d}}\left( {\frac{{\rho h^{3} }}{\eta }\frac{{dp_{h} }}{dx}} \right)}}{dx} = 12u_{s} \frac{d(\rho h)}{{dx}} $$

where *ρ* is density, *P*_*h*_ is oil film pressure, *η* is viscosity, Pa·s.

**Figure 17:**
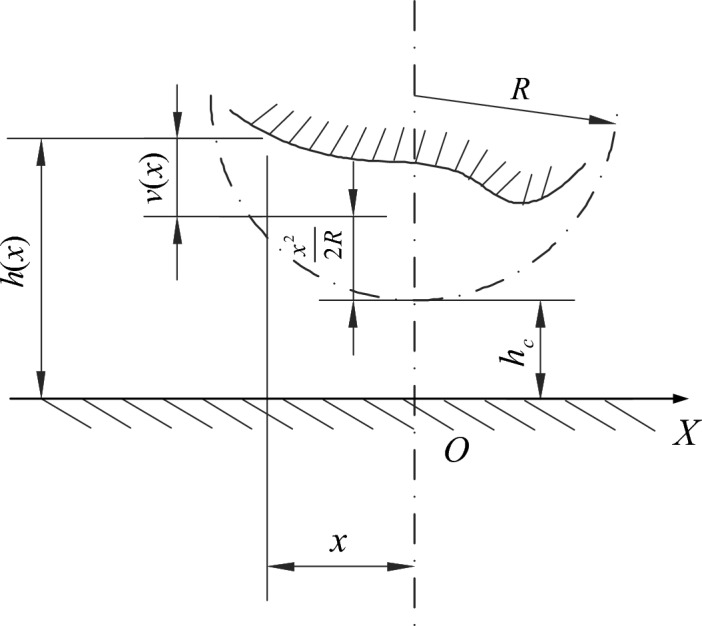
Oil film thickness calculation diagram.

In order to calculate the parameters in the lubrication model, the film thickness equation, viscosity equation and density equation need to be brought into the Reynolds equation for iterative solution. These formulas are as follows:

Film thickness equation:

The calculation formula of oil film thickness is composed of two parts: the basic oil film thickness and the elastic deformation, as shown in the Fig. 17. So the film thickness equation is given by:

4 $$ h({\text{x}}) = h_{c} + \frac{{x^{2} }}{2R} + v(x) $$

where *h* is film thickness, *V* is elastic deformation, *R* is equivalent cylindrical contact radius.

Visco-pressure equation:

5 $$ \eta = \eta_{{_{0} }} \exp \left\{ {(\ln \eta_{0} + 9.67)\left[ { - 1 + \left( {1 + {\raise0.7ex\hbox{${p_{h} }$} \!\mathord{\left/ {\vphantom {{p_{h} } {p_{0} }}}\right.\kern-0pt} \!\lower0.7ex\hbox{${p_{0} }$}}} \right)^{z} } \right]} \right\} $$

where *η*_*0*_ is initial viscosity, *P*_*0*_ is initial oil film pressure.

Density–pressure equation:

6 $$ \rho = \rho_{0} \left( {1 + \frac{{0.6p_{h} }}{{1 + 1.7p_{h} }}} \right) $$

where *ρ*_*0*_ is initial density.

### Asperity contact model

The load in the contact area under mixed lubrication is shared by the oil film pressure and the asperity contact pressure. In this study, the asperity contact model established by Greenwood and Tripp is used^28^. It is assumed that the asperity is isotropic and its height obeys the Gaussian distribution. When the two surfaces are in contact, the asperity contact pressure is a function of the film thickness ratio, and the contact pressure *P*_a_ is:

7 $$ P_{a} (h) = \frac{16\sqrt 2 }{{15}}\pi (n\beta \sigma )^{2} \sqrt {\frac{\sigma }{\beta }} \cdot E \cdot F_{2.5} (\lambda ) $$

8 $$ F_{2.5} (\lambda ) = \left\{ {\begin{array}{*{20}l} {4.4086 \times 10^{ - 5} (4 - \lambda )^{6.804} ,} & {\quad \lambda \le 4} \\ {0,} & {\quad \lambda > 4} \\ \end{array} } \right. $$

In the formula, *n* is the asperity density, m^−2^, *β* is the radius of curvature, m, respectively, take *nβσ* = 0.04; take *σ/β* = 10^−3^; *E* is the comprehensive elastic modulus, Pa.

### Calculation of friction

According to Formula (2), the load *W* consists of two items: the asperity contact load *W*_*a*_ and oil film load* W*_L_. Similarly, the friction force is composed of two items: the friction force generated by the shear stress in the hydrodynamic oil film and the friction force generated by the asperity contact.

9 $$ F_{{\text{f}}} = \int {\tau_{f} } ({\text{x}}){\text{dx}} $$

10 $$ F_{c} = \int {f_{c} } \times p_{a} ({\text{x}}){\text{dx}} $$

The oil film shear stress is solved by the Eyring model^29^:

11 $$ \tau_{f} ({\text{x}}) = \tau_{0} \arcsin {\text{h}}\left( {{\raise0.7ex\hbox{${\eta \gamma }$} \!\mathord{\left/ {\vphantom {{\eta \gamma } {\tau_{0} }}}\right.\kern-0pt} \!\lower0.7ex\hbox{${\tau_{0} }$}}} \right) $$

where *τ*_0_ is the Eyring stress, Pa,* γ* is the shear rate, 1/s,* η* is lubricant dynamic viscosity, Pa·s.

It is assumed that when the film thickness ratio λ ≥ 4, the asperities will not contact. When λ < 4 but the boundary film is not broken, the friction coefficient of the boundary film *µ*_a_ is usually between 0.005 and 0.2^29^. When the boundary film breaks, the friction coefficient will increase sharply to the dry friction level, the friction coefficient is set to 0.5^30^. The friction coefficient can be expressed as the following piecewise function:

12 $$ f_{c} = \left\{ {\begin{array}{*{20}l} 0 & {\quad \lambda \ge 4} \\ {\mu_{a} } & {\quad \lambda < 4\;\; \, and\;\; \, p_{a} < S(B,T,\tau )} \\ {0.5} & {\quad \lambda < 4 \, \;\;and\;\; \, p_{a} \ge S(B,T,\tau )} \\ \end{array} } \right. $$

### Solution process and preliminary verification

**Figure 18:**
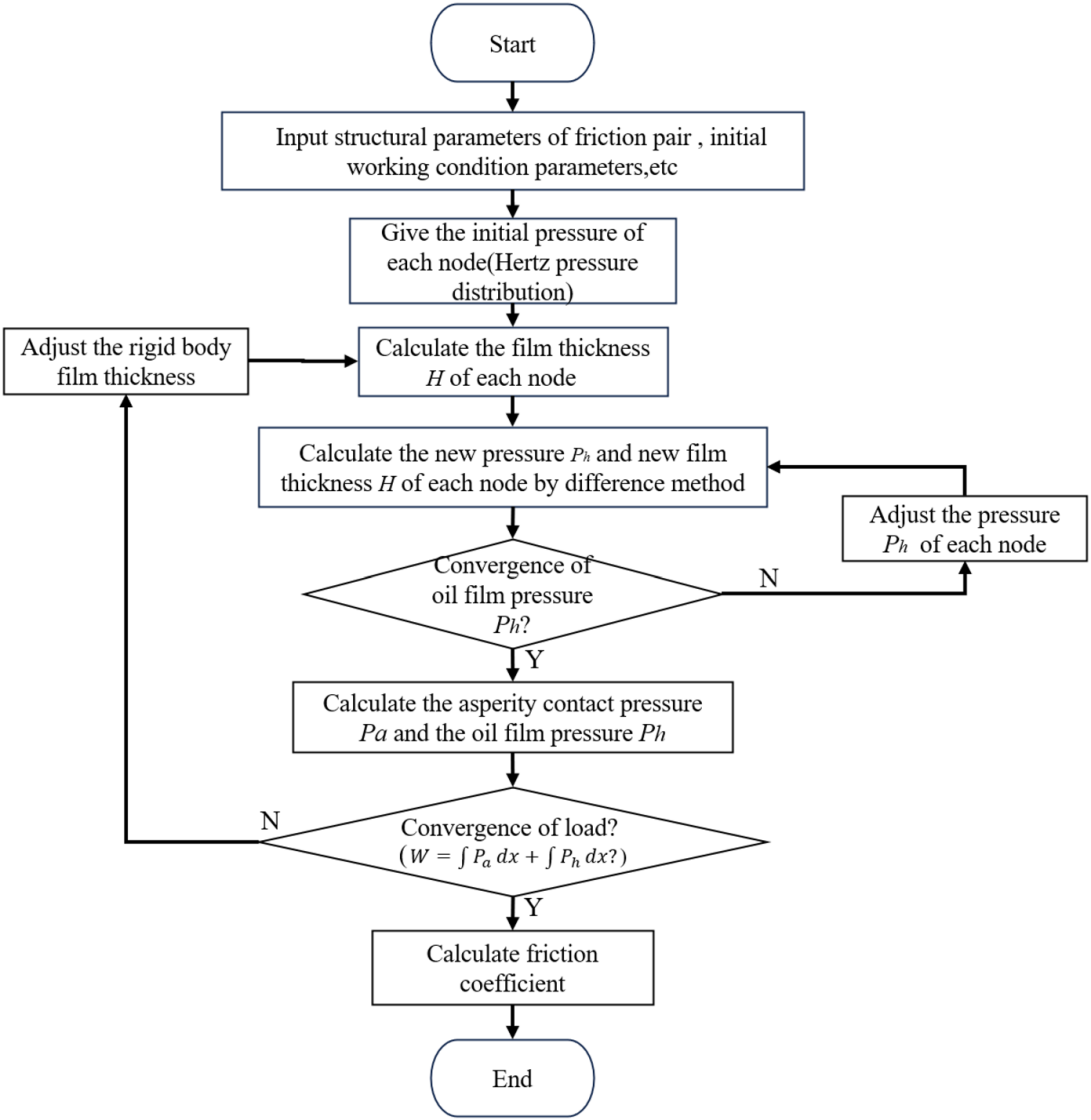
Flow chart of mixed lubrication calculation program.

In the process of solving the model, the asperity model (G–T model) is used to solve the asperity contact pressure Pa^28^, and the Reynolds model is used to solve the oil film pressure Ph and film thickness *H*^9^*.* The criterion for the end of the iterative process is to determine whether the given load* W* is equal to the integral sum of the asperity contact pressure *P*_*a*_ and the oil film pressure *P*_*h*_. Due to the calculation of viscosity and film thickness is related to pressure, the general approach is to give an initial pressure distribution (Hertz pressure distribution) to calculate the film thickness and viscosity value. The calculated film thickness is brought into the asperity contact pressure to obtain the asperity contact pressure under the film thickness. Then, the film thickness and viscosity values are substituted into the Reynolds equation to solve for the new distribution of oil film pressure. The previous pressure distribution is then iteratively modified, and the elastic deformation and film thickness are recalculated. This process is repeated until the pressure difference between two consecutive iterations becomes very small and satisfies the load balance condition. When the iteration finishes, the iteration is over, getting the final pressure distribution and the film thickness of elastic deformation. The program flow chart is illustrated in Fig. 18.

**Figure 19:**
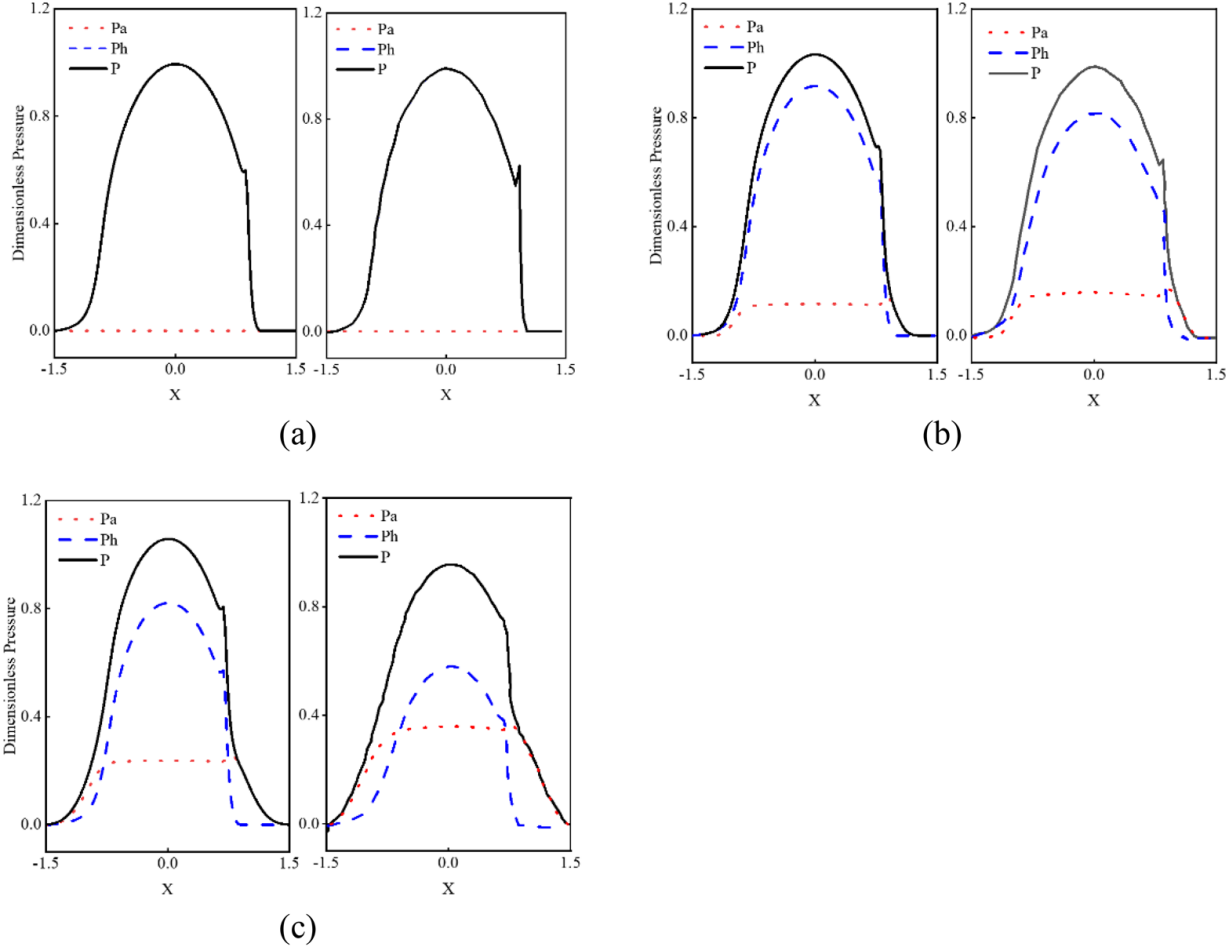
Effect of surface roughness on pressure distribution (the left one is calculated according to this model, the right one is the results in Ref.^20^).

To verify the correctness of the above line contact mixed lubrication mode, the simulation results of this model are compared with the calculation results of the infinite line contact mixed lubrication model established by Masjedi^31^. In the simulation, the same dimensionless and input parameters are used, namely load parameters, material parameters and velocity parameters. In Fig. 19, the right one is the result of the simulation using the model in this paper, the right one is the result of simulation using the model in Ref.^20^. Figures 18a, 19b,c respectively show the pressure distribution between surfaces with different dimensionless roughness of 5 × 10^−6^ μm, 2 × 10^−5^ μm and 5 × 10^−5^ μm. It can be seen that the results calculated by the two models are consistent; with the increase of roughness, the proportion of asperity bearing pressure in the total pressure increases gradually. The model in Ref.^31^ analyzes the elastohydrodynamic lubrication problem of infinite long line contact, while this paper analyzes the elastohydrodynamic lubrication problem of finite long line contact. Because the axial rough peak texture of finite long line contact will greatly increase the thickness of oil film, which makes the oil film parameters larger, so the asperity contact pressure *P*_*a*_ is larger.

### Experimental verification method

**Figure 20:**
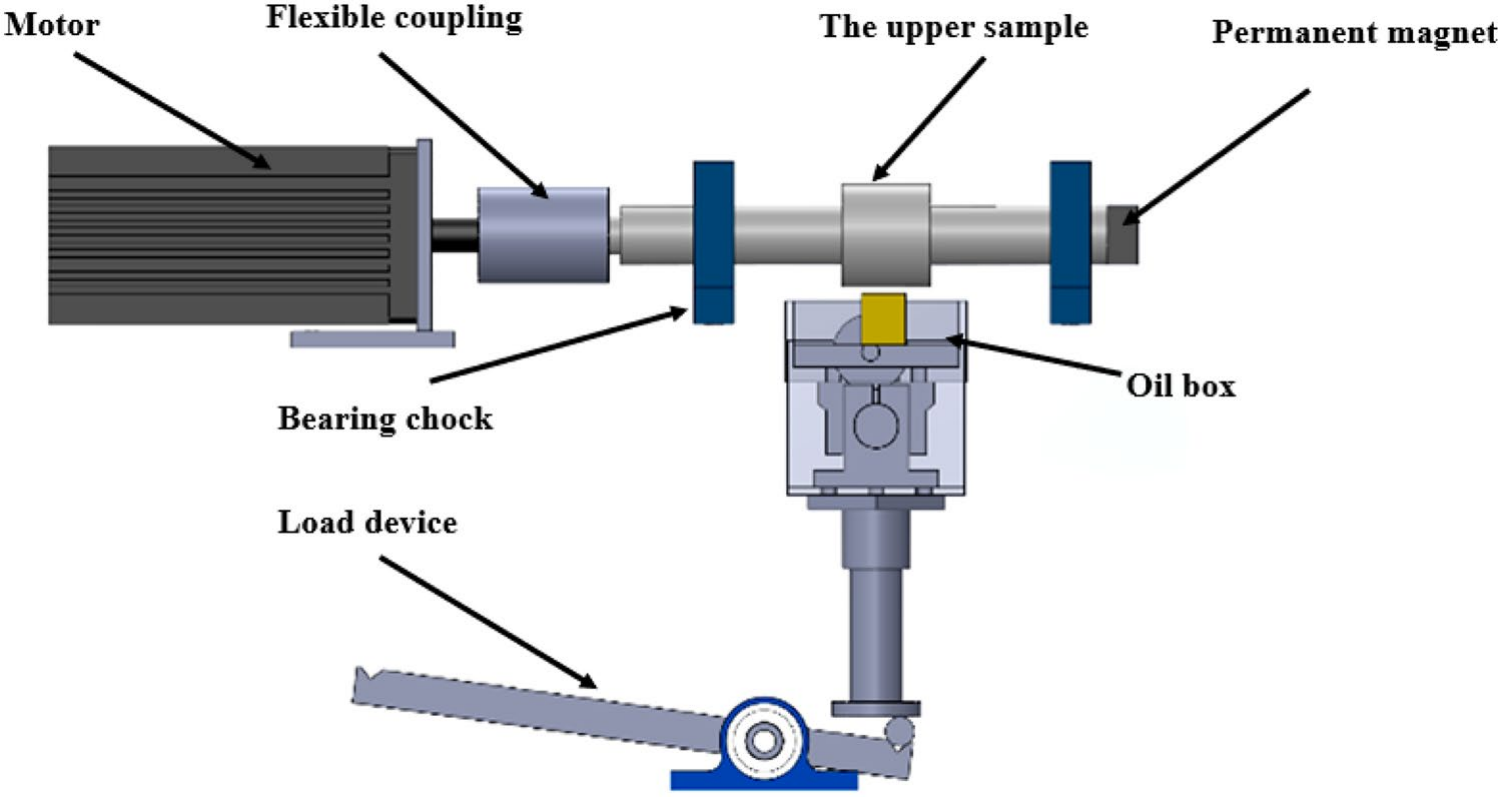
Schematic diagram of line contact wear test machine.

The rationality of simulation results is verified by tribology test. Figure 20 is the self-made line contact wear test machine. Table 5 gives the related parameters in the experiment. The upper sample is a 45-steel stepped shaft, and the lower sample is a brass block. The lubricant used in the verification experiment is CF10W-40 lubricating oil with magnetic liquid ([bmim][FeCl_4_]) as additive, and the verification experiment is carried out at 55 °C oil temperature, 0.1 m/s rotation speed and 0.1 T magnetic field intensity. The magnetic field strength is provided by the permanent magnet at the right end of the step shaft, and the control method of the magnetic field strength is the same as that of section “Test equipment and test preparation”.

**Table 5:** Experimental parameters.

Parameter	Value
Load, *W*	100–1200 N
Speed, *N*	0.4 m/s
Bearing radius, *R*	40 mm
Width, *b*	15 mm
Equivalent elastic modulus, *E*	110 GPa
Surface roughness, *σ*	0.59 μm
Ambient temperature, *T*_0_	55 °C
Lubricant viscosity, *η*_0_	0.061 Pa·s

**Figure 21:**
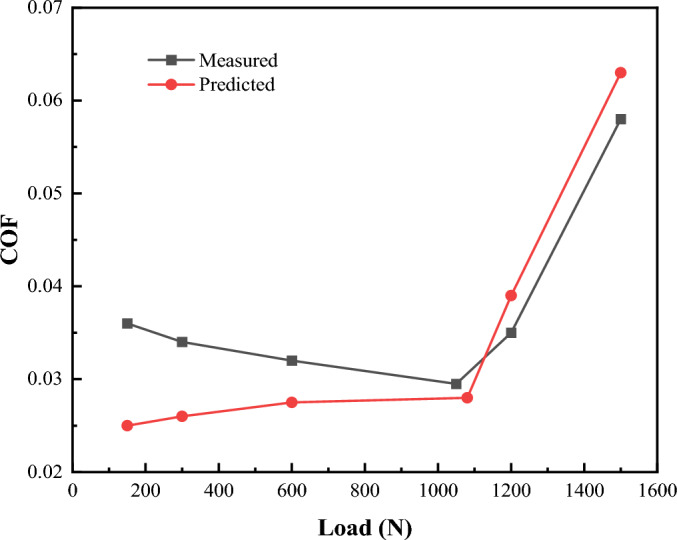
Comparison of theory and experiments.

Figure 21 shows the comparison between the test results and the simulation results. By contrast, it can be found that the actual measured friction coefficient and the simulated friction coefficient show a similar trend, which confirms that the mixed lubrication model proposed in this paper can accurately reflect the tribological characteristics. In the experimental test, when the load is small, the friction pair is in a fluid lubrication state. Due to the increase of the load, more heat will be generated, which will lead to the decrease of the viscosity of the lubricant and the decrease of the viscous resistance in the contact area. However, the model calculation does not consider the influence of the heat generated during the contact process on the model, so it is different from the actual measured value. Some small errors may be due to the change of surface texture and roughness height in the experiment, and these factors are not considered in the model.

## Application of mixed lubrication model

### The influence of sliding speed

**Figure 22:**
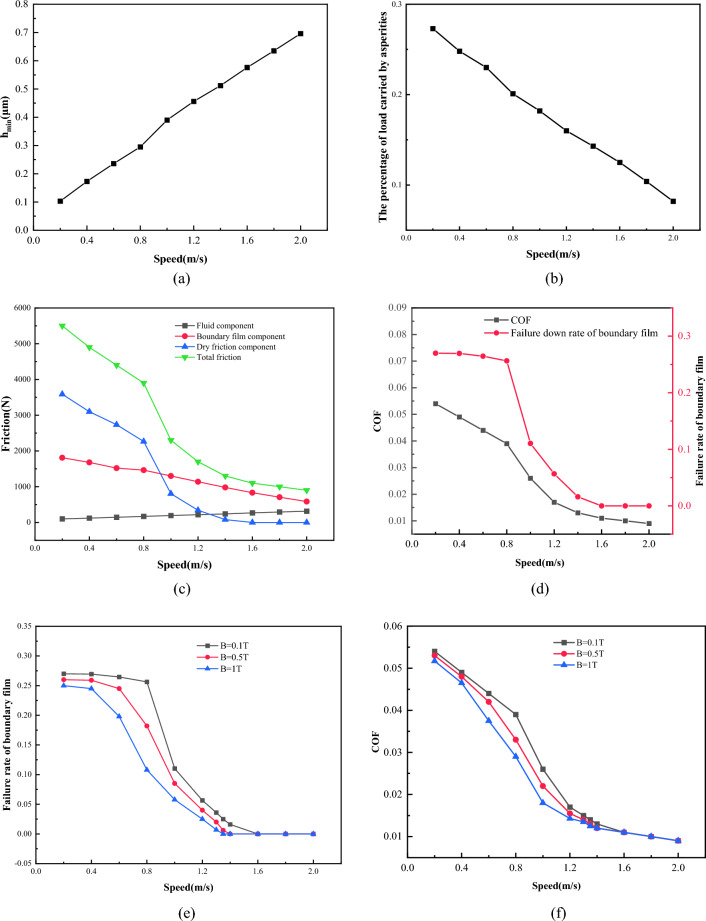
Influence of sliding speed on lubrication performance.

Figure 22 shows the minimum film thickness and friction coefficient curves under the conditions of loading *W* = 1 × 10^5^ N/m, temperature *T* = 55 °C, viscosity* η* = 0.06 Pa·s, roughness *σ* = 0.7 μm and rotation speed changing from 0.2 to 2.0 m/s. It can be seen from Fig. 22a, that as the speed increases, the minimum oil film thickness *h*_*min*_ increases from about 0.1–0.7 μm. As the increase of rotational speed, the area of asperity contacts decreases from 27 to 8%, as shown in Fig. 22b. The variation of each friction component with speed under mixed lubrication is shown in Fig. 22c. When the speed is low, the dry friction component accounts for a large proportion due to the rupture of a large number of boundary films. As the increase of velocity, the dry friction component decreases continuously, and the total friction force is dominated by the boundary film component and the fluid component. As shown in Fig. 22d, when the speed rises to 1.6 m/s, the failure rate of boundary film is reduced to 0, and the lubrication state changes. There is no longer local dry friction, and the lubrication state changes into a mixed lubrication dominated by boundary lubrication and fluid lubrication. Figure 22e,f, respectively show the failure rate of boundary film and friction coefficient with speed under 0.1 T, 0.5 T and 1 T magnetic field strength. It can be found that due to the effect of magnetic field strength on the strength of the boundary film, the larger magnetic field strength makes the boundary film failure rate of the friction pair smaller and the friction coefficient lower. When the speed is small, although with the increase of speed, the oil film thickness will increase continuously, which will make the friction pair in a better lubrication environment, but according to the boundary film strength model in section “Boundary film strength model”, the increase of shear stress will also lead to the failure of the boundary film. Therefore, when the speed is small, the speed has little effect on the failure rate of the boundary film. This also leads to the magnetic field conditions have little effect on the friction coefficient. According to the analysis, it can be seen that too low speed makes the lubrication state of the friction pair poor, which easily leads to the failure of the boundary film. Properly increasing the speed is conducive to forming a good lubrication state, reducing friction, and improving mechanical efficiency and lubrication performance.

### The influence of lubricant viscosity

**Figure 23:**
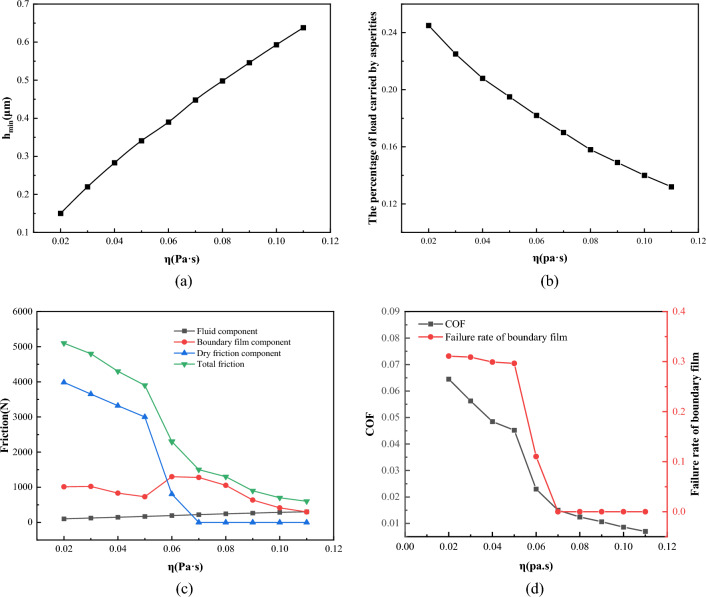
Influence of viscosity on lubrication performance.

Figure 23 shows the effect of different viscosities *η* on the lubrication performance when the load* W* = 1 × 10^5^ N/m, the temperature* T* = 55 °C, the roughness *σ* = 0.7 μm, and the speed *V* = 1 m/s. Figure 23a shows that as the viscosity changes, the minimum oil film thickness of the lubricating oil increases from 0.15 to 0.67 μm. It can be seen from Fig. 23b that the percentage of load carried by asperities is reduced from 24.5 to 12.2%. Figure 23c shows the curve of the friction component with viscosity. When the viscosity is low, the viscosity is too low to generate an effective lubricating oil film, so the dry friction component and the boundary film component account for a large proportion. As the increase of viscosity, the dry friction component decreases continuously. When the viscosity increases to 0.07 Pa·s, there is almost no dry friction component. It can be seen from Fig. 23d that when the viscosity is less than 0.05 Pa·s, the failure rate of boundary film is close to 30%, the friction coefficient is higher than 0.04, and the lubrication state is mainly dry friction and boundary lubrication. As the viscosity increases, the failure rate of the boundary film begins to decrease. When the viscosity is higher than 0.07 Pa·s, the failure rate of the boundary film is almost 0, and the lubrication state changes to a mixed lubrication dominated by fluid lubrication. So, it can be seen that increasing the viscosity of lubricating oil appropriately can make it easier to form an oil film between friction pairs, achieve a good lubrication effect, and then improve the service life of friction pairs.

### The influence of roughness

**Figure 24:**
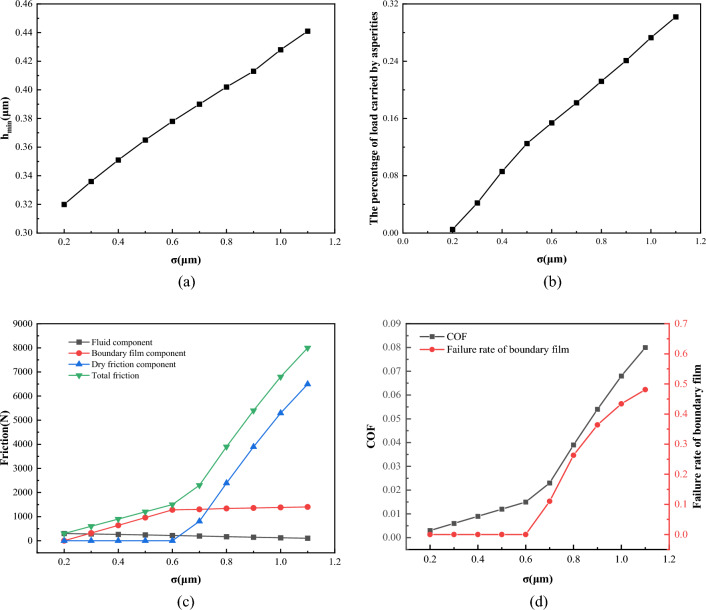
Influence of roughness on lubrication performance.

Figure 24 shows the effect of different surface roughness on lubrication performance when load *W* = 1 × 10^5^ N/m, temperature *T* = 55 °C, viscosity *η* = 0.06 Pa·s, and speed *V* = 1 m/s. It can be seen from Fig. 24a,b that as the increase of surface roughness, the minimum oil film thickness increases from 0.32 to 0.44 μm, and the percentage of load by asperities increases from almost 0 to 30%. Due to the increase of roughness, the asperity carries a larger proportion of the load, which reduces the oil film pressure and increases the oil film thickness. In Fig. 24c, it can be found that the friction force increases with the increase of roughness. When the roughness is below 0.6 μm, the dry friction component is almost zero. When the roughness increases to more than 0.6 μm, the dry friction component rises sharply and its proportion in the friction force increases gradually. Excessive roughness will lead to the failure of the boundary film, and as the roughness further increases, the failure rate of the boundary film also increases. As shown in Fig. 24d, when the roughness increases from 0.6 to 1.2 μm, the boundary film failure rate increases from almost 0 to 50% which induces a rapid increase in the friction coefficient. It can be seen that the roughness of the contact surface has a significant effect on the lubrication performance of the friction pair. Therefore, optimizing the surface roughness of the friction pair can greatly improve the lubrication performance of the friction pair^16^.

### The influence of load

**Figure 25:**
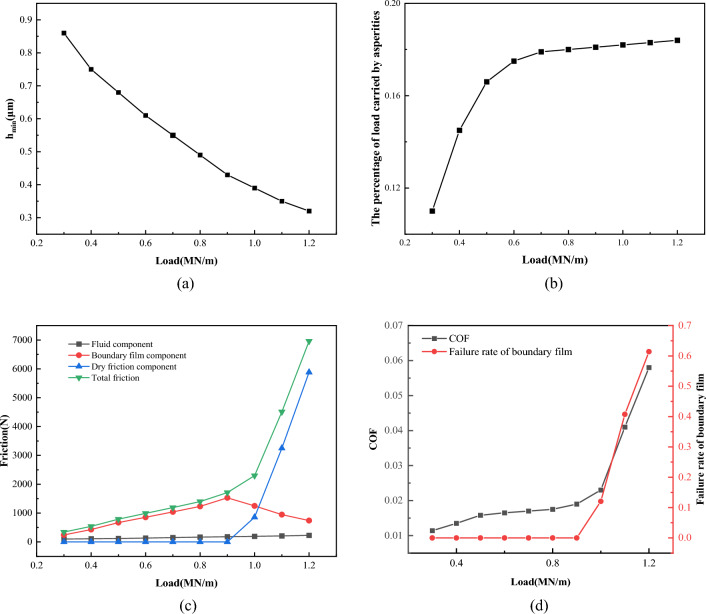
Influence of load on lubrication performance.

Figure 25 shows the effect of different load on lubrication performance when the roughness *σ* = 0.7 μm, temperature *T* = 55 °C, viscosity *η* = 0.06 Pa·s, and speed *V* = 1 m/s. Figure 25a,b shows that as the load increases, the minimum oil film thickness decreases from 0.86 to 0.3 μm, and the percentage of load carried by asperity increases from 11 to 18%. Figure 25c reflects the changing trend of friction and its components in the process of load increase. Compared with Fig. 25d, it can be seen that when the load is less than 0.9 MN/m, the boundary film has not failed. As the load increases, the fluid component and the boundary film component increase continuously, while the dry friction component is almost zero. When the load is further increased to 0.9 MN/m, the boundary film component of the friction gradually decreases due to the failure of the boundary film, and the friction is mainly provided by dry friction. At this time, the friction and friction coefficient increase sharply, and the lubrication state of the friction pair begins to be dominated by dry friction. Therefore, selecting the appropriate load range plays a vital role in maintaining good lubrication of the friction pair.

### The influence of temperature

**Figure 26:**
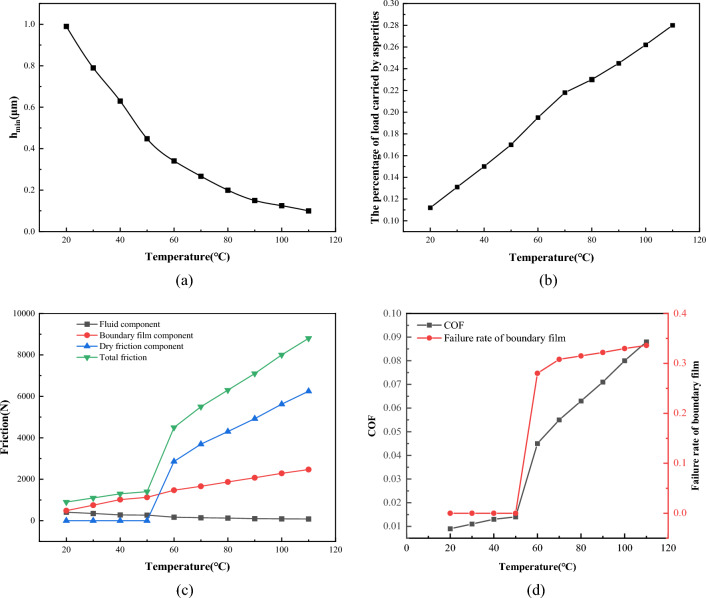
Influence of temperature on lubrication performance.

It is necessary to explore the effect of temperature because it affects both the oil viscosity and the boundary film strength. Figure 26 shows the effect of different temperature on the lubrication performance when the load* W* = 1 × 10^5^ N/m, viscosity *η* = 0.06 Pa·s, roughness *σ* = 0.7 μm, and speed *V* = 1 m/s. From Fig. 26a, it can be seen that the minimum oil film thickness. From Fig. 26a, it can be seen that the minimum oil film thickness is about 0.99 μm when the temperature is 20 °C, when the temperature rises to 110 °C, the minimum oil film thickness decreases to 0.09 μm. When the temperature rises, the viscosity of the lubricating oil decreases, which makes the lubricating oil film between the friction pairs become thinner, which also leads to an increasing percentage of load carried by asperity. When the temperature rises to 120 °C, the percentage of load carried by asperity increases to 28%, as shown in Fig. 26b. It can be seen in Fig. 26c that the variation curve of each friction component as the increase in temperature. When the temperature rises to 50 °C, the boundary film begins to fail, which makes the dry friction component begin to increase sharply. When the temperature rises to 110 °C, the dry friction component accounts for about 70% of the total friction component. As shown in Fig. 26d, when the temperature is lower than 50 °C, the failure rate of boundary film is almost 0, and the friction coefficient is less than 0.15. As the increase of temperature, the boundary film begins to fail, and the friction coefficient begins to increase sharply. When the temperature rises to 110 °C, the friction coefficient rises to 0.09, and the failure rate of boundary film reaches 34%. Therefore, the heat dissipation of the lubricating oil should be controlled during the operation of the friction pair, because the excessive oil temperature will reduce the strength of the boundary film and make the lubrication state worse.

### The influence of magnetic field strength

**Figure 27:**
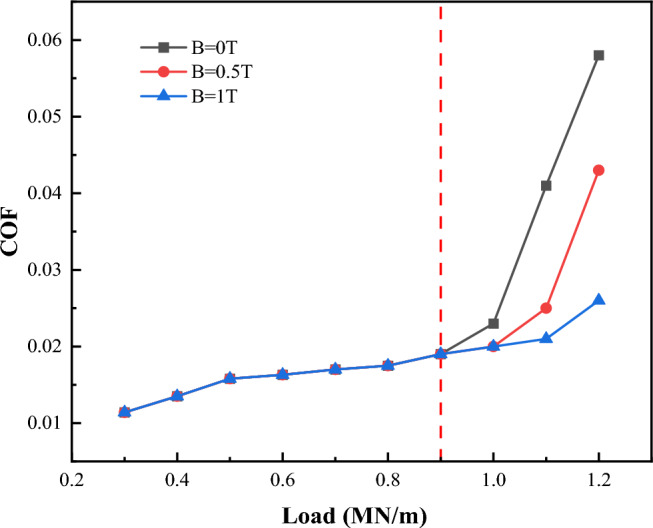
Influence of magnetic field strength on lubrication performance.

Due to the strength of the boundary film being related to the strength of the magnetic field, the influence of the magnetic field strength on the lubrication performance should also be considered in the analysis of this paper. Figure 27 is the variation of friction coefficient with load under different magnetic field strengths. The working conditions are as follows: roughness *σ* = 0.7 μm, temperature *T* = 55 °C, viscosity *η* = 0.06 Pa·s, speed *V* = 1 m/s, and load *W* varies from 0.3 to 1.2 MN/m. From the analysis of section “Solution process and preliminary verification”, it can be seen that as the load reaches a certain strength, the failure of the boundary film will occur, which will lead to a sudden increase in the friction coefficient. It can be seen from Fig. 27 that when the load is 1.1 MN/m, the friction coefficient under the condition of no magnetic field has increased to 0.04, while the friction coefficient under the condition of 1 T magnetic field is only 0.02. This is because the boundary film almost does not fail under the condition of 1 T magnetic field. Therefore, an appropriate amount of magnetization on the friction pair is beneficial to improve the lubrication performance of the friction pair.

## Conclusions

1. Under the condition of magnetic field, the use of magnetic fluid additives can improve the anti-friction and anti-wear properties of lubricating oil by generating a boundary film with higher strength. An oil-soluble magnetic ion solution [bmim][FeCl_4_] is used as a lubricating oil additive. Its anti-friction and anti-wear effect are verified by experiments, and the anti-friction and anti-wear effect are better under the action of the magnetic field. When [bmim][FeCl_4_] was used as an additive to configure a magnetic lubricant with a mass fraction of 2%, the friction coefficient decreased by about 20%, the wear depth decreased by about 45%, and the anti-friction effect and anti-wear effect were further improved under 0.1 T applied magnetic field strength, which were 10% and 20%, respectively.
2. The test results show that magnetic field strength, film thickness, shear stress, and temperature are the key factors affecting the strength of boundary film. This paper developed a boundary film strength model considering magnetic field strength, which can be used to predict the change of lubrication state of magnetic fluid lubricating oil under different magnetic field conditions, which is beneficial to engineering applications.
3. A mixed lubrication model considering the boundary film strength is established. Compared with the traditional mixed lubrication model based on oil film thickness, the model based on the strength of boundary film can be used to reflect the mixed lubrication characteristics under different anti-wear additives because it can directly reflect the failure principle of boundary film. The model can predict the change of lubrication state, and effectively reflect the minimum oil film thickness, asperity bearing rate, failure rate of boundary film, friction coefficient and the action law of fluid, boundary film and dry friction component in friction force under different working conditions. The calculated friction coefficient is basically consistent with the experimental results.
4. Through the analysis of the lubrication performance of the line contact friction pair under mixed lubrication, it can be seen that the lubrication performance of the friction pair can be improved by appropriately increasing the rotational speed and the viscosity of the lubricating oil. When the rotational speed is higher than 1.6 m/s or the viscosity is higher than 0.07 Pa·s, the failure rate of boundary film is almost 0, and the friction coefficient is less than 0.02. Selecting the appropriate load and ensuring sufficient heat dissipation of the friction pair is an important condition to maintain the good lubrication performance of the friction pair. When the load is higher than 1.1 MN/m or the temperature is higher than 100 °C, the failure rate of boundary film is higher than 30%, and the dry friction component accounts for more than 65% of the total friction force, resulting in a sudden increase in the friction coefficient. Improve the surface roughness of the friction pair. Increasing the strength of the boundary film can significantly increase the critical load of the friction pair. Applying a certain strength of the magnetic field to the magnetic liquid as a lubricant can effectively increase the strength of the boundary film. When 1 T magnetic field strength is applied, the critical load that the boundary film can withstand is increased by 20%.

## Data Availability

The data that support the findings of this study are available from the corresponding author, Z.Y., upon reasonable request.
